# Korean Chestnut Honey Suppresses HSV-1 Infection by Regulating the ROS–NLRP3 Inflammasome Pathway

**DOI:** 10.3390/antiox12111935

**Published:** 2023-10-30

**Authors:** Eun-Bin Kwon, Young Soo Kim, Buyun Kim, Se-Gun Kim, Sung-Joon Na, Younghoon Go, Hong Min Choi, Hye Jin Lee, Sang Mi Han, Jang-Gi Choi

**Affiliations:** 1 Korean Medicine Application Center, Korea Institute of Oriental Medicine, Daegu 41062, Republic of Korea; 2 Department of Agricultural Biology, National Institute of Agricultural Sciences, Rural Development Administration, Wanju 55365, Republic of Korea; 3 Special Forest Resources Division, National Institute of Forest Science, Suwon 16631, Republic of Korea; nsj10@forest.go.kr

**Keywords:** HSV-1, KCH, ROS, NF-кB, mitochondria, NLRP3 inflammasome

## Abstract

Herpes simplex virus 1 (HSV-1) is double-stranded DNA virus that belongs to the Orthoherpesviridae family. It causes serious neurological diseases of the central nervous system, such as encephalitis. The current U.S. Food and Drug Administration (FDA)-approved drugs for preventing HSV-1 infection include acyclovir (ACV) and valacyclovir; however, their long-term use causes severe side effects and often results in the emergence of drug-resistant strains. Therefore, it is important to discover new antiviral agents that are safe and effective against HSV-1 infection. Korean chestnut honey (KCH) has various pharmacological activities, such as antioxidant, antibacterial, and anti-inflammation effects; however, antiviral effects against HSV-1 have not yet been reported. Therefore, we determined the antiviral activity and mechanism of action of KCH after HSV-1 infection on the cellular level. KCH inhibited the HSV-1 infection of host cells through binding and virucidal steps. KCH decreased the production of reactive oxygen species (ROS) and calcium (Ca^2+^) following HSV-1 infection and suppressed the production of inflammatory cytokines by inhibiting nuclear factor kappa-light-chain-enhancer of activated B cells (NF-кB) activity. Furthermore, we found that KCH inhibited the expression of the nod-like receptor protein 3 (NLRP3) inflammasome during HSV-1 infection. Taken together, the antiviral effects of KCH occur through multiple targets, including the inhibition of viral replication and the ROS-mediated NLRP3 inflammasome pathway. Our findings suggest that KCH has potential for the treatment of HSV-1 infection and related diseases.

## 1. Introduction

Herpes simplex virus (HSV) is a prevalent and complex DNA virus with two types: HSV-1 and HSV-2 [1]. According to the World Health Organization (WHO), around 67% of people under age 50 are infected with HSV-1, which causes oral herpes, while HSV-2, the main cause of genital herpes, affects about 13% of individuals aged 15–49 [2,3].

HSV infections vary widely in severity, with some individuals experiencing mild or even asymptomatic cases and others suffering from painful recurring outbreaks. Although antiviral medications can help manage symptoms and reduce outbreak frequency, there is currently no cure for HSV. The virus periodically reactivates from a dormant state and causes recurrent sores or blisters, which tend to resolve on their own. These reactivations occur in specific areas depending on the HSV type. In immunocompromised individuals, reactivation can lead to severe complications such as central nervous system infections [1,2,3,4].

Recently, several studies have focused on the association between HSV-1 infection and reactive oxygen species (ROS). ROS regulate signaling pathways that activate the immune response against viruses, in particular, DNA sensing pathways [5]. In the case of herpes viruses, ROS not only enhance replication, but also induce virus reactivation from latency and potentially contribute to virally induced cancers [6]. In addition, HSV-1 infection activates the excessive inflammatory response through viral proteins, endoplasmic reticulum (ER) stress, mitochondria dysfunction, ROS generation, aberrant ion concentrations, and protein aggregates [7,8]. Viral infection induces mitochondrial dysfunction, disrupts mitochondrial dynamic proteins, and alters the intracellular physiological environment through post-translational modification and gene expression regulation [8,9]. Mitochondria also control cell signaling pathways and are intimately involved in the host inflammatory response. Mitochondria can release reactive oxidative species (mtROS) to activate the nod-like receptor protein 3 (NLRP3) inflammasome [10].

The NLRP3 inflammasome plays an important role in the development and progression of inflammation-related diseases and is the main inflammatory sensor activated by various stimuli [11,12]. The NLRP3 inflammasome consists of three major components, NLRP3, apoptosis-associated speck-like protein containing a CARD (ASC), and precursor caspase-1, which drive sterile inflammation during various pathologies [11,12,13]. Upon the activation of the inflammasome, precursor caspase-1 is cleaved into caspase-1, which further cleaves and activates downstream proinflammatory cytokines, such as interleukin-1β (IL-1β) and IL-18 [13]. Inflammation can be exacerbated by the overproduction of proinflammatory cytokines like IL-1β and IL-18, which are induced by inflammasome activation. This overproduction is a crucial aspect of the host’s innate immune response [14,15]. Therefore, the NLRP3 inflammasome is an important therapeutic target for HSV replication and HSV infection-associated diseases [16,17].

At present, there are nucleoside analogs, such as acyclovir (ACV) and valacyclovir, which are U.S. Food and Drug Administration (FDA)-approved for the treatment of HSV infection and HSV encephalitis, with high cure rates. However, long-term use of ACV often results in serious side effects and the emergence of drug-resistant virus strains. In addition, these nucleoside analogs are not effective against latent infecting viruses because of their mechanical properties [18]. Therefore, safe and effective antiviral agents against HSV-1 are needed. Natural products play a crucial role in the development of antiviral drugs due to their established safety. We recently reported the effectiveness of treatment against HSV-1 based on natural products. Our studies have shown that natural products, such as *Quercus acuta* Thunb. (Fagaceae), *Vaccinium bracteatum* Thunb, and *Mori ramulus*, inhibit the replication of HSV-1 by regulating intracellular ROS. In addition, research has reported reduced HSV-1-induced inflammation responses such as nuclear factor kappa-light-chain-enhancer of activated B cells (NF-кB) pathway [19,20,21]. Undoubtedly, managing HSV-1 infection and addressing ROS-mediated inflammation represent significant therapeutic objectives for HSV-1. Therefore, our aim was to confirm the antiviral treatment effect of honey by targeting the regulation of the ROS–NLRP3 inflammasome complex in HSV-1 infection.

The honey used in this study was Korean chestnut honey (KCH), which is Korea’s representative summer honey produced next to acacia honey. KCH has a dark brown color and a bitter taste. It has excellent antioxidant and antibacterial properties and contains various nutrients, such as amino acids, minerals, and vitamins [22]. Previously, we reported the antiviral effect of chestnut honey against influenza virus [23]; however, the antiviral effect of KCH against HSV is unknown. Therefore, we examined the antiviral effect of KCH against HSV. In addition, we examined the mechanism of KCH against ROS and NLRP3 inflammasome induced by HSV-1 infection.

## 2. Materials and Methods

### 2.1. Materials

Dulbecco’s modified Eagle’s medium (DMEM), fetal bovine serum (FBS), and antibiotic–antimycotic were obtained from Hyclone (Pittsburgh, PA, USA). Enhanced chemiluminescence (ECL), polyvinylidene fluoride (PVDF), 2,7-dichlorofluorescein diacetate assay (DCFH-DA), Flou-4, MitoSOX, and Rhod-2 were purchased from Thermo Fisher Scientific (Waltham, MA, USA). PRO-prep protein extraction solution, 10× Tris-glycine sodium dodecyl sulfate (SDS) buffer, and 10× transfer buffer were purchased from Intron Biotechnology (Seoul, Korea). DMSO and 3-(4,5-dimethylthiazol-2-yl)-2,5-diphenyltetrazolium bromide (MTT) were purchased from Sigma-Aldrich (St. Louis, MO, USA). IL-6, TNF-α, and IL-1β ELISA kits were purchased from BD Biosciences.

### 2.2. Korean Chestnut Honey

**Figure 1:**
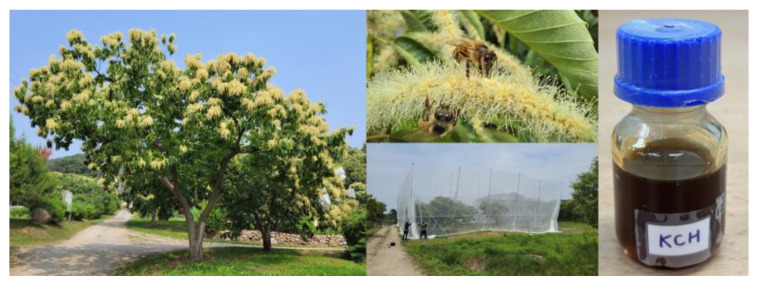
Korean chestnut honey.

KCH (Figure 1) was provided by Dr. Sung-Joon Na of the Special Forest Resources Division, National Institute of Forest Science.

### 2.3. Cells and Viruses

The Vero African green monkey kidney cell line was obtained from the American Type Culture Collection (Manassas, VA, USA). Vero cells were grown in DMEM containing 10% fetal bovine serum (FBS) and 1% antibiotic–antimycotic and maintained at 37 °C in a CO_2_ incubator. The HSV-1 strains (KBPV-VR-733), green-fluorescent protein (GFP)-encoding HSV strains, and HSV-2 strains (KBPV-VR-53) were stored at −80 °C and were thawed on ice before use [19,20].

### 2.4. Plaque Assay

For virus quantification, a plaque assay was used as previously described [19,20]. Briefly, the cells were seeded on 12-well plates at 3 × 10^5^ cells/well. The cells were infected with HSV-1 (MOI = 0.1) and HSV-2 (MOI = 1) at 37 °C for 2 h and washed twice with PBS. Three different concentrations of KCH (1.25, 2.5, and 5 mg/mL) and ACV (30 μmol/L) were grown on a 1.5% agarose gel for 4 days. After reacting, fixing with 4% paraformaldehyde (PFA), and staining with 1% crystal violet solution for 2 h, the detecting plaques corresponding to each group were imaged and the plaque numbers were quantified.

### 2.5. Cell Survival Assay

Cell viability was measured using the crystal violet method according to the manufacturer’s instructions. Vero cells were seeded into 24-well plates at 1 × 10^5^ cells/well, infected with HSV-1 and HSV-2 for 2 h. The medium was replaced with fresh medium and incubated with KCH. After 48 h, the dark violet formazan produced in cells was solubilized with 100% MeOH, and the absorbance at 600 nm was measured using an Epoch Microplate Reader (BioTek, Washington, DC, USA).

### 2.6. Evaluation of Antiviral Effects

The cells were infected with GFP-encoding HSV-1 (MOI = 2) for 2 h and then treated with various concentrations of KCH (1.25, 2.5, and 5 mg/mL) and 30 μmol/L ACV for 48 h. The levels of viral-GFP were measured using fluorescence microscopy (Nikon ECLIPSE Ti-U, Nikon Co., Tokyo, Japan) and a CytoFLEX flow cytometer (Beckman Coulter Inc., Pasadena, CA, USA).

### 2.7. Time-of-Addition Assay

To understand the steps by which KCH inhibits HSV-1, we divided the virus pretreatment protocol into three steps (entry, binding, and virucidal) based on how it can be blocked during host infection. This protocol was performed as described previously [24].

### 2.8. Western Blot Analysis

Cells were lysed using PRO-PREP protein extract solution and quantitated using the Bradford method. Nuclear and cytoplasmic proteins were isolated using nuclear isolation kits (Thermo), according to the manufacturer’s instructions. Equal amounts of protein samples were separated by 8–15% of SDS-polyacrylamide gel electrophoresis (SDS-PAGE) and transferred to PVDF membranes. They were blocked using 0.5× Ez-Block Chemi (Amherst, MA, USA), and then the membranes were reacted with various antibodies and detection was evaluated using a ChemiDoc imaging system (UVITEC, Cleaver Scientific Ltd., England, UK) with an ECL reagent (Thermo Scientific, Rockford, IL, USA).

### 2.9. Fluorescence Analysis

ROS and calcium were induced by HSV-1 infection and were detected via flow cytometry using DCFH-DA, Flou-4, MitoSOX, and Rhod-2. To perform the assay, Vero cells (1 × 10^5^ cells/well) were seeded into 24-well plates. After 2 h of infection with HSV-1, the cells were washed with DPBS without Ca^2+^ and Mg^2+^, and a new complete medium was added. After incubation for 48 h with various concentrations of KCH, 10 μmol/L DCFH-DA, Flou-4, MitoSOX, and Rhod-2, the final concentration treated was 1 μmol/L in wells. The mixtures were incubated for an additional 30 min. Also, mitochondrial membrane potential (MMP, Δψm) was analyzed using 40 nmol/L DioC6(3). Fluorescence was detected using a CytoFLEX flow cytometer (Beckman Coulter Inc., Pasadena, CA, USA).

### 2.10. Antioxidant-Related Enzyme Activity

The activations of catalase (CAT) and superoxide dismutase (SOD) in cell lysates were measured using CAT and SOD assays (Cayman), respectively, according to the manufacturer’s instructions.

### 2.11. Immunofluorescence Staining

The cells were fixated with 4% paraformaldehyde (PFA) for 10 min at 25 °C and washed three times with PBS. Fixated cells were blocked with 5% BSA for 30 min, followed by phosphorylated NF-κB antibodies overnight at 4 °C. Next, they were washed with PBS-T and incubated with Alexa-fluor 488 conjugated antibody for 2 h on a rocker. Finally, after washing, they were stained with Hoechst 33342 for 15 min. Cultured wells were added to mounting solution and visualized using a fluorescence microscope (Nikon Corporation, Tokyo, Japan).

### 2.12. Cytokines

Interleukin (IL)-6, tumor necrosis factor (TNF)-α (mouse), and IL-1β levels in supernatants were determined using ELISA antibody kits (BD) based on the manufacturer’s instructions.

### 2.13. Statistical Analysis

The experiments are expressed as the mean ± standard error of mean (SEM). The significance of differences in mean values between treated and untreated groups were calculated using a one-way ANOVA. Tukey’s post hoc test was used for multigroup comparisons. The analyses were performed using GraphPad PRISM^®^ Version 8.01 (GraphPad, Boston, MA, USA) software and *p*-values < 0.05 were considered statistically significant.

## 3. Results

### 3.1. Antiviral Effects of KCH on HSV-1 and HSV-2 Infected in Vero Cells

**Figure 2:**
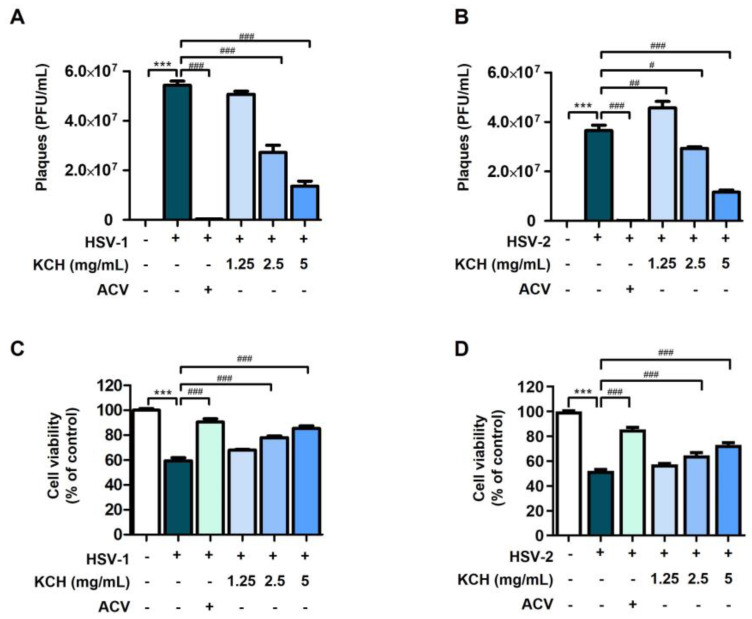
KCH inhibits HSV-1 and HSV-2 replication in Vero cells. The cells were infected with HSV-1 or HSV-2 for 2 h and treated with KCH (1.25, 2.5, and 5 mg/mL) or ACV (30 μmol/L) for 48 h. ACV was used as a positive control. After incubation, the virus was quantitated using a plaque assay for (**A**) HSV-1 and (**B**) HSV-2. Cytotoxicity induced by (**C**) HSV-1 and (**D**) HSV-2 was measured by the crystal violet method. Bar graph (mean ± SEM) statistical data were measured using a one-way analysis of variance with Tukey’s post hoc test. *** *p* < 0.001 compared with the untreated group. ### *p* < 0.001; ## *p* < 0.01 and # *p* < 0.05 compared with the HSV-1-infected group.

We determined the antiviral effect of KCH against HSV-1 and HSV-2 in Vero cells. Prior to this experiment, we confirmed that treatment with KCH alone did not affect the viability of Vero cells (Supplementary Figure S1). The antiviral effect of KCH against HSV-1 and/or HSV-2 was confirmed by plaque and cytotoxicity assays. As shown in Figure 2A,B, KCH inhibited viral replication by HSV-1 and HSV-2 in a dose-dependent manner. Also, KCH effectively restored the cell viability reduced by HSV-1 and/or HSV-2 infection (Figure 2C,D). These data indicate that KCH restores more of the reduced cell growth resulting from HSV-1 infection compared with HSV-2. Therefore, we confirmed that KCH significantly inhibits the infection of Vero cells by HSV-1 and HSV-2.

**Figure 3:**
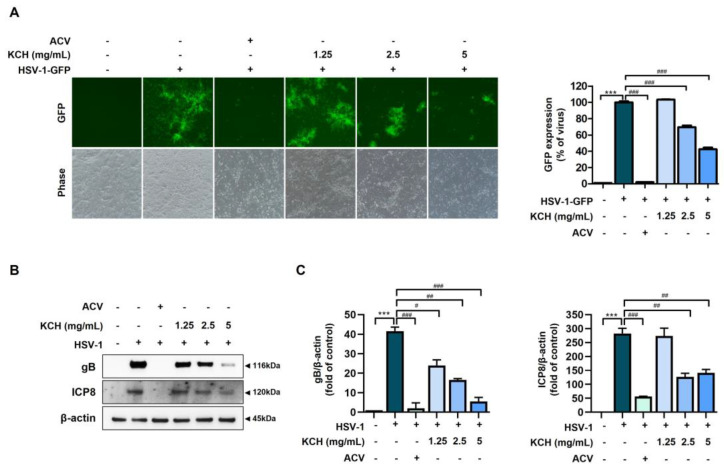
KCH suppresses the infection of Vero cells by HSV-1. The cells were infected with GFP encoding HSV-1 for 2 h and treated with KCH (1.25, 2.5 and 5 mg/mL) or ACV (30 μmol/L) for 48 h. (**A**) GFP was detected using fluorescence microscopy and flow cytometry. Scale bar = 100 μm. (**B**) Western blot analysis of the HSV viral proteins, gB and ICP8, and (**C**) quantitative analysis of the protein bands using ImageJ software. Three independent experiments were performed. Bar graph (mean ± SEM) statistical data were determined using a one-way analysis of variance with Tukey’s post hoc test. *** *p* < 0.001 compared with the untreated group. ### *p* < 0.001; ## *p* < 0.01 and # *p* < 0.05 compared with the HSV-1-infected group.

We observed viral inhibition by KCH in Vero cells infected with GFP-encoding HSV-1 (HSV-1-GFP) via fluorescence microscopy and flow cytometry. Various concentrations of KCH reduced the expression of HSV-1 GFP (Figure 3A). The expression of gB and ICP8, which are HSV viral proteins, was assessed by Western blot analysis. KCH significantly reduced the expression of gB and ICP8 (Figure 3B). These data indicate that KCH restores cell viability after HSV-1 exposure and inhibits infection by the HSV virus.

### 3.2. KCH Inhibited HSV-1 Binding and Virucidal Activity

**Figure 4:**
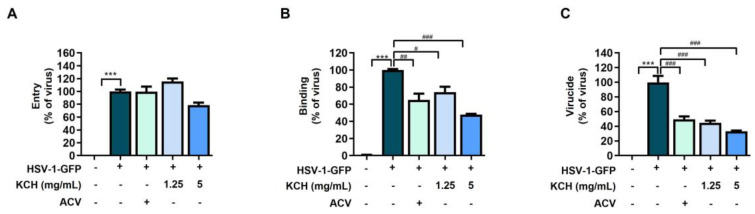
Effect of KCH on the time-of-addition assay. (**A**) Following the entry assay. (**B**) Following the binding assay. (**C**) Following the virucide assay. Bar graph (mean ± SEM) statistical data were determined using a one-way analysis of variance with Tukey’s post hoc test. *** *p* < 0.001 compared with the untreated group. ### *p* < 0.001; ## *p* < 0.01 and # *p* < 0.05 compared with the HSV-1-infected group.

To determine which viral infection phase is targeted by KCH during HSV-1 infection, we evaluated viral infection at entry, binding, and virucidal sites in the presence of KCH. As shown in Figure 4A–C, KCH inhibited the binding and virucide stage of HSV-1 but did not affect the entry site. These results indicate that the inhibition of HSV-1 binding could stem from damage caused by KCH to virus particles prior to their interaction with host cells.

### 3.3. KCH Ameliorates Mitochondrial Dysfunction in Vero Cells Caused by HSV-1 Infection

Reactive oxygen species (ROS) and calcium (Ca^2+^) play important roles as second messengers in signaling pathway activation [25]. HSV infections stimulate other cellular processes, such as the production of ROS and Ca^2+^ [26]. To determine how these radicals are involved in HSV recognition, we examined whether HSV infection induces the formation of cellular ROS and Ca^2+^.

**Figure 5:**
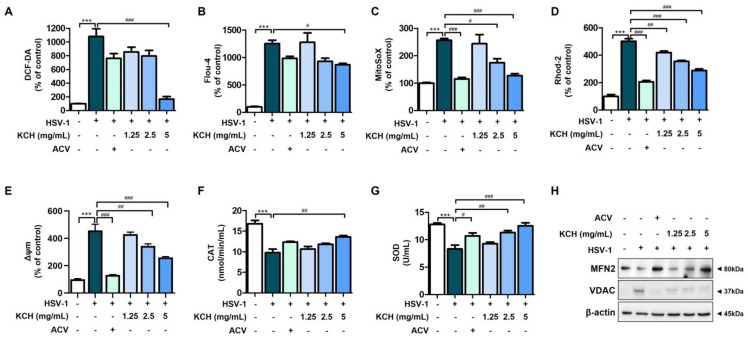
KCH reduced infection of HSV-1-induced mitochondrial stress in Vero cells. Intracellular ROS and Ca^2+^ were measured using (**A**) DCF-DA and (**B**) Fluo-4AM. Mitochondrial ROS and Ca^2+^ were determined using the (**C**) MitoSoX and (**D**) Rhod-2 staining methods. (**E**) Mitochondrial membrane potential (MMP, Δψm) was measured using DioC6(3). All fluorescence values were detected by flow cytometry. (**F**) CAT and (**G**) SOD activities were measured using an antioxidant enzyme assay kit. (**H**) The expressions of MFN2 and VDAC were detected via Western blot analysis. Bar graph (mean ± SEM) statistical data were determined using a one-way analysis of variance with Tukey’s post hoc test. *** *p* < 0.001 compared with the untreated group. ### *p* < 0.001; ## *p* < 0.01 and # *p* < 0.05 compared with the HSV-1-infected group.

We infected HSV and then measured intracellular ROS and Ca^2+^ using the fluorescent reagents, DCF-DA and Fluo-4, respectively. As shown Figure 5A,B, KCH reduced intracellular ROS and Ca^2+^ levels following HSV infection. In addition, the data confirmed that ROS and Ca^2+^ in mitochondria, which were excessively produced following HSV-1 infection, were decreased by KCH treatment (Figure 5C,D). These results indicate that KCH significantly reduces the ROS and Ca^2+^ levels in the cytoplasm and mitochondria that had increased following HSV-1 infection. Mitochondrial membrane potential (MMP, ΔΨ m) is associated with the production of ROS and Ca^2+^. Therefore, we determined whether KCH reduces MMPs, which are increased by HSV-1 infection. The results indicated that KCH decreased MMP expression following viral infection (Figure 5E). Moreover, KCH increased the activity of antioxidant enzymes, including catalase (CAT) and superoxide dismutase (SOD), which were reduced by HSV-1 infection in Vero cells (Figure 5F,G).

Mitofusin 2 (MFN2) is an outer mitochondrial membrane protein that promotes mitochondrial fusion and is important for mitochondrial and endoplasmic reticulum function. Low MFN2 causes both mitochondrial dysfunction and ROS release [27]. Also, mitochondrial voltage-dependent anion channels (VDAC) mediate the uptake of released Ca^2+^ into the mitochondria, resulting in mitochondrial Ca^2+^ overload and increased mtROS [28,29]. Therefore, as a result of confirming the expression levels of MFN2 and VDAC via Western blot analysis, KCH increased the expression of the MFN2 protein, which was decreased by HSV-1 infection, whereas KCH decreased the expression of VDAC, which was increased by HSV-1 infection (Figure 5H). Taken together, these results suggest that KCH restores mitochondrial dysfunction, including ROS and Ca^2+^ production, caused by HSV-1 infection, and increases antioxidant enzyme activity.

### 3.4. KCH Reduced Inflammation Response in HSV-1-Infected Vero Cells

**Figure 6:**
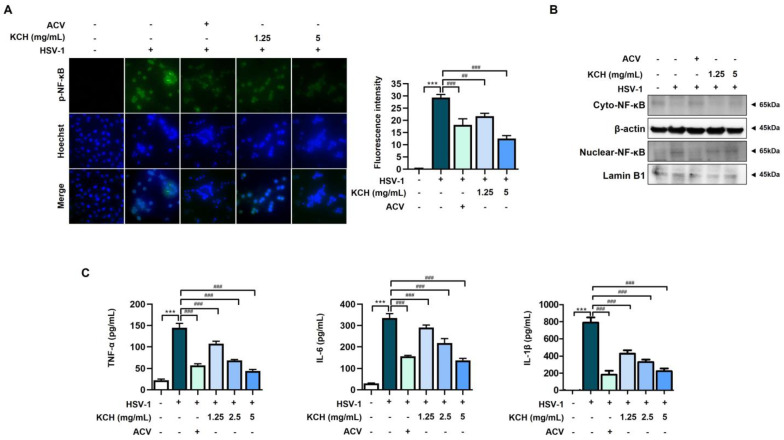
KCH decreased infection and HSV-1- induced phosphorylation of NF-κB and inflammatory cytokines. Vero cells were infected with HSV-1 for 2 h and treated with KCH (1.25, 2.5, and 5 mg/mL) or ACV (30 μmol/L) for 48 h. After the reaction, (**A**) staining for p-NF-κB determined via the IF method, and also (**B**) cytosol and nuclear isolation via the nuclear isolation kit for the expression of p-NF-κB were performed. Scale bar = 100 μm (**C**) levels of TNF-α, IL-6, and IL-1β were measured using an ELISA kit. Bar graph (mean ± SEM) statistical data were calculated using one-way analysis of variance with Tukey’s post hoc test. *** *p* < 0.001 compared with the untreated group. ### *p* < 0.001 and ## *p* < 0.01 compared with the HSV-1-infected group.

Previous studies have shown that the activation of NF-κB is one of the key mechanisms occurring in HSV-1-infected cells and is closely linked to ROS production [30]. Therefore, we determined whether KCH inhibits the translocation of NF-κB in HSV-1-increased Vero cells by immunofluorescence analysis. As shown in Figure 6A, the phosphorylation of NF-κB was translocated to the nucleus following HSV-1 infection, whereas migration was inhibited by KCH. Also, as a result of confirming the expression of p-NF-κB by analyzing nuclear and cytoplasmic proteins, we found that KCH reduced the translocation of p-NF-κB to the nucleus, which was increased following HSV-1 infection (Figure 6B). The results indicate that KCH inhibits the translocation of NF-kB to the nucleus and suppresses p-NF-kB protein expression in the nucleus.

Activation of NF-kB subsequently leads to increased levels of inflammatory cytokines, such as interleukin (IL)-1β, IL-6, IFN, and TNF-α [31]. We determined whether KCH suppresses the production of inflammatory cytokines, including interleukins, TNF-α, IL-6, and IL-1β. As shown in Figure 6C, KCH decreased the production of inflammatory cytokines, which were increased by HSV-1 infection in Vero cells. Therefore, the results indicate that KCH regulates the inflammation response following HSV-1 infection.

### 3.5. KCH Inhibits the Expression of the NLRP3 Inflammasome following HSV-1 Infection

**Figure 7:**
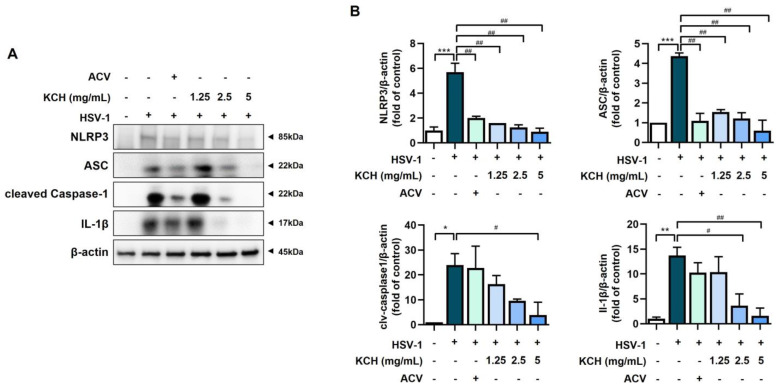
KCH suppresses infection of HSV-1-induced expression of NLRP3 inflammasome. (**A**) Expression of the NLRP3 inflammasome, including ACS, caspase-1, and IL-1β, was analyzed via Western blot. (**B**) Quantification of protein bands using the ImageJ software. Bar graph (mean ± SEM) statistical data were determined using one-way analysis of variance with Tukey’s post hoc test. *** *p* < 0.001; ** *p* < 0.01 and * *p* < 0.05 compared with the untreated group. ## *p* < 0.01 and # *p* < 0.05 compared with the HSV-1-infected group.

NLRP3 inflammasome activation is a highly regulated process for controlling the secretion of potent inflammatory cytokines, such as IL-1β and IL-18, which are essential during bacterial infection and the inflammatory response [32]. In addition, ROS regulates the activation of the NLRP3 inflammasome [33]. We found that components of the NLRP3 inflammasome complex, including NLRP3, ACS, and caspase-1, were decreased at the protein level by KCH in HSV-1-infected cells. In addition, KCH suppressed the expression of IL-1β, which increased following HSV-1 infection (Figure 7A,B). Therefore, KCH reduces the expression of the NLRP3 inflammasome complex, which is increased by viral infection.

## 4. Discussion

Various honeys, including manuka, tualang, and *Tilia amurensis* honey, and associated compounds, such as methylglyoxal (MGO), have attracted attention as effective natural therapies because of their ability to attenuate acute inflammation by enhancing the immune response. Several studies have demonstrated antiviral effects against HIV, influenza virus, and varicella-zoster virus [34,35]. Among these, a study on *Tilia amurensis* honey has reported on the protective effect of influenza A virus though the activation of interferon beta signaling and the interferon inducible transmembrane protein 3 (IFITM3) pathway [35].

Recent studies have highlighted the link between HSV-1 infection and reactive oxygen species (ROS), which play a role in immune activation against viruses [5,8,9]. HSV-1 can exploit ROS to enhance replication, reactivate from latency, and potentially contribute to virus-induced cancers. Additionally, HSV-1 triggers excessive inflammation through various mechanisms, including mitochondrial dysfunction and NLRP3 inflammasome activation. The NLRP3 inflammasome is a key player in inflammation-related diseases, consisting of NLRP3, ASC, and caspase-1, which produce proinflammatory cytokines. Targeting the NLRP3 inflammasome holds promise for controlling HSV replication and related diseases [10,11,12,13,14,15,16,17].

**Figure 8:**
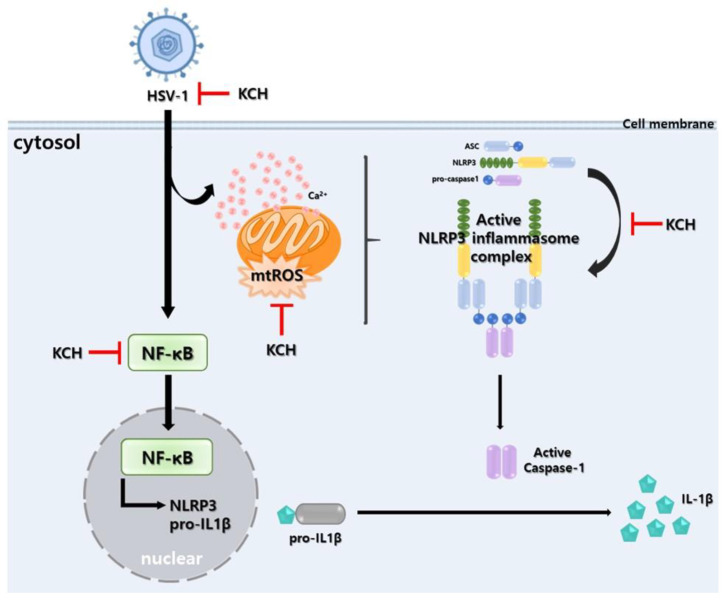
Antiviral mechanism of KCH upon HSV-1 infection in Vero cells. KCH attenuates viral infection by suppressing ROS-mediated NLRP3 inflammasome after HSV-1 infection.

In the present study, we demonstrated, for the first time, that KCH inhibits HSV replication and the ROS-mediated NLRP3 inflammasome, which is increased in HSV-1-infected cells (Figure 8). KCH inhibits viral infection by disrupting viral binding and exhibiting increased virucidal effects. In addition, we confirmed that KCH significantly reduced intracellular ROS and calcium, which were increased by viral infection. The connection between calcium channels and virus replication is a captivating field of recent research. Calcium ions (Ca^2+^) play a critical role in various cellular processes, including those related to viral replication [36]. Viral infection also increases the release of calcium from the ER, which enters through the VDAC for uptake into the mitochondria [26,37]. The expression of VDAC is reduced by KCH treatment; however, it is necessary to confirm the openness of VDAC, not only by expressing it as a channel in mitochondria, but also by measuring its electrical stimulation.

Furthermore, increased calcium and ROS increase the secretion of reactive oxygen species from the mitochondria, resulting in increased activation of the NLRP3 inflammasome. Previous studies indicate that the NLRP3 inflammasome is closely associated with the inflammatory response and affects HSV viral replication. We showed that KCH suppresses the NLRP3 inflammasome complex in HSV-1-infected cells. HSV-1 infection activates the cGAS-STING pathway, which stimulates the intracellular immune response and viral replication [38,39,40]. Of note, upon HSV-1 infection, STING binds to NLRP3, promotes the NLRP3-ASC interaction as an indicator of the inflammasome complex assembly, and facilitates NLRP3-mediated ASC oligomerization, which is an important step for inflammasome activation [40]. However, because the effect of KCH on STING was not confirmed in this study, it should be evaluated through additional experiments. Thus, the regulation of the NLRP3 inflammasome may represent a major target in controlling latent infection and frequent inflammation by the HSV-1 virus. KCH can potentially reduce HSV-1-induced encephalitis by regulating HSV-1 infection and HSV-1 infection-induced inflammation through NLRP3 inflammasome regulation.

We are also investigating compounds in KCH that exhibit anti-HSV effects. Among these compounds, kynurenic acid (KYNA) is a quinoline alkaloid specifically found in chestnut honey that serves as an indicator of chestnut honey and is a potential marker for identification. In a previous study, we reported the anti-influenza effects of kynurenic acid (KYNA), a specific compound in chestnut honey [23]. However, we did not examine KYNA in relation to HSV-1. Therefore, we investigated the antiviral effect of KYNA on HSV-1 infection. However, KYNA did not have a significant effect. Thus, we speculate that various components in KCH other than KYNA may contribute to antiviral efficacy against HSV1. We plan to conduct further studies to analyze the various components present in KCH and study individual compounds with potential anti-HSV effects.

The long-term use of HSV-1 agents, such as ACV, results in the emergence of drug-resistant mutant strains in addition to adverse side effects. Therefore, new drugs are needed as an anti-HSV-1 strategy based on novel targets and reinforcement of the existing drug paradigm. Our findings indicate that KCH is effective against HSV-1 infection. The antiviral effect of KCH suggests the effectiveness of multi-targeting through the suppression of viral replication and the ROS-mediated NLRP3 inflammasome pathway. However, it is believed that further research on the ingredients responsible for the antiviral effects of KCH is necessary. Nevertheless, KCH may have potential as a new treatment for HSV-1 infection and related diseases.

## 5. Conclusions

In this study, we demonstrated the antiviral effect of KCH against HSV-1 in Vero cells. In addition, KCH reduced mitochondrial dysfunction as evidenced by decreased intracellular/mitochondrial ROS and Ca^2+^ levels that are induced by HSV-1-infected cells. Furthermore, NF-κB activation and the NLRP3 inflammasome were increased following HSV-1 infection, but were decreased following KCH treatment. Therefore, KCH suppresses HSV-1 infection by regulating the ROS-mediated NLRP3 inflammasome pathway.

## Data Availability

All the data are available within the article and Supplementary Materials.

## Supplementary Materials

The following supporting information can be downloaded at https://www.mdpi.com/article/10.3390/antiox12111935/s1, Figure S1: Effect of KCH on cell viability.
