# Supplementary material for: Korean Chestnut Honey Suppresses HSV-1 Infection by Regulating the ROS–NLRP3 Inflammasome Pathway
Source: Antioxidants (Basel). 2023 Oct 30;12(11):1935. doi: 10.3390/antiox12111935 (PMC10669648; doi:10.3390/antiox12111935)
Supplement: Supplementary file 1 [file antioxidants-12-01935-s001.zip › antioxidants-2646029-supplementary.pdf]

Supplementary figures

Korean Chestnut Honey Suppresses HSV-1 Infection by Regulating the ROS–NLRP3 Inflammasome Pathway

Eun-Bin Kwon [1], Young Soo Kim [1], Buyun Kim [1], Se-Gun Kim [2], Sung-Joon Na [3], Younghoon Go [1], Hong Min Choi [2], Hye Jin Lee [2], Sang Mi Han [2] and Jang-Gi Choi [1,*]

[1] Korean Medicine Application Center, Korea Institute of Oriental Medicine, Daegu 41062, Republic of Korea
[2] Department of Agricultural Biology, National Institute of Agricultural Sciences, Rural Development Administration, Wanju 55365, Republic of Korea
[3] Special Forest Resources Division, National Institute of Forest Science, Suwon 16631, Republic of Korea; nsj10@forest.go.kr
* Correspondence: Correspondence: jang-gichoi@kiom.re.kr; Tel.: +82-53-940-3865

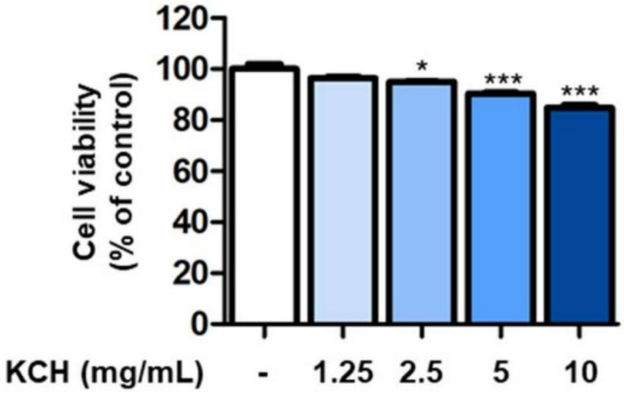

Figure S1. Effect of KCH on cell viability. Vero cells were treated with various concentrations of CH for 48 h. Cell viability was assessed using an MTT assay. The experiments were performed three times independently, *** $p < 0.001$, * $p < 0.05$.
